# The hitchhiker's guide to Europe: the infection dynamics of an ongoing *Wolbachia* invasion and mitochondrial selective sweep in *Rhagoletis cerasi*

**DOI:** 10.1111/mec.13571

**Published:** 2016-03-15

**Authors:** Hannes Schuler, Kirsten Köppler, Sabine Daxböck‐Horvath, Bilal Rasool, Susanne Krumböck, Dietmar Schwarz, Thomas S. Hoffmeister, Birgit C. Schlick‐Steiner, Florian M. Steiner, Arndt Telschow, Christian Stauffer, Wolfgang Arthofer, Markus Riegler

**Affiliations:** ^1^ Department of Forest and Soil SciencesBoku, University of Natural Resources and Life SciencesHasenauerstr. 381190ViennaAustria; ^2^ Faculty of Science and TechnologyFree University of Bozen‐BolzanoUniversitätsplatz 139100Bozen‐BolzanoItaly; ^3^ Department of Biological SciencesGalvin Life Sciences Building, University of Notre DameNotre DameIN 46556USA; ^4^ Center for Agricultural Technology AugustenbergNesslerstr. 23‐3176227KarlsruheGermany; ^5^ Department of Crop Sciences, BokuUniversity of Natural Resources and Life SciencesPeter‐Jordan‐Str. 821190ViennaAustria; ^6^ Government College UniversityAllama Iqbal RoadFaisalabad38000Pakistan; ^7^ School of Biological SciencesUniversity of QueenslandSt LuciaQLD 4072Australia; ^8^ Department of BiologyWestern Washington University510 High StreetMS 9160BellinghamWA98225USA; ^9^ Institute of EcologyFaculty Biology/ChemistryUniversity of BremenLeobener Str. NW2B4040, 28359BremenGermany; ^10^ Institute of EcologyUniversity of InnsbruckTechnikerstr. 256020InnsbruckAustria; ^11^ Institute for Evolution and BiodiversityWestfalian Wilhelms‐University MünsterHüfferstr. 148149MünsterGermany; ^12^ Hawkesbury Institute for the EnvironmentWestern Sydney UniversityLocked Bag 1797Penrith, NSW2751Australia

**Keywords:** endosymbiont, horizontal transmission, infection dynamics, modelling, selective sweep

## Abstract

*Wolbachia* is a maternally inherited and ubiquitous endosymbiont of insects. It can hijack host reproduction by manipulations such as cytoplasmic incompatibility (CI) to enhance vertical transmission. Horizontal transmission of *Wolbachia* can also result in the colonization of new mitochondrial lineages. In this study, we present a 15‐year‐long survey of *Wolbachia* in the cherry fruit fly *Rhagoletis cerasi* across Europe and the spatiotemporal distribution of two prevalent strains, *w*Cer1 and *w*Cer2, and associated mitochondrial haplotypes in Germany. Across most of Europe, populations consisted of either 100% singly (*w*Cer1) infected individuals with haplotype HT1, or 100% doubly (*w*Cer1&2) infected individuals with haplotype HT2, differentiated only by a single nucleotide polymorphism. In central Germany, singly infected populations were surrounded by transitional populations, consisting of both singly and doubly infected individuals, sandwiched between populations fixed for *w*Cer1&2. Populations with fixed infection status showed perfect association of infection and mitochondria, suggesting a recent CI‐driven selective sweep of *w*Cer2 linked with HT2. Spatial analysis revealed a range expansion for *w*Cer2 and a large transition zone in which *w*Cer2 splashes appeared to coalesce into doubly infected populations. Unexpectedly, the transition zone contained a large proportion (22%) of *w*Cer1&2 individuals with HT1, suggesting frequent intraspecific horizontal transmission. However, this horizontal transmission did not break the strict association between infection types and haplotypes in populations outside the transition zone, suggesting that this horizontally acquired *Wolbachia* infection may be transient. Our study provides new insights into the rarely studied *Wolbachia* invasion dynamics in field populations.

## Introduction

Heritable endosymbionts play an important role in the ecology and evolution of animals (McFall‐Ngai *et al*. 2013). The alphaproteobacterium *Wolbachia* infects a broad range of arthropods and filarial nematodes and is probably the most common endosymbiont (Werren *et al*. 2008). It is mostly maternally inherited and spreads by increasing reproductive fitness of infected females (Engelstädter & Hurst 2009). The most common phenotype of reproductive manipulation by *Wolbachia* is the induction of cytoplasmic incompatibility (CI; e.g. Hoffmann & Turelli 1997) that results in embryonic death in matings between infected males and uninfected females, while reciprocal matings are compatible. This reproductive advantage of *Wolbachia* infected over uninfected females enhances the spread of *Wolbachia* within a population (Turelli & Hoffmann 1991). However, in certain cases *Wolbachia* can be retained within a population with little or no reproductive manipulation (e.g. Hamm *et al*. 2014). Although *Wolbachia* is one of the best studied endosymbionts, only few studies describe its spatial dynamics in field populations (Turelli & Hoffmann 1991; Jaenike *et al*. 2006; Narita *et al*. 2006; Hoffmann *et al*. 2011; Kriesner *et al*. 2013; Schuler *et al*. 2013; Atyame *et al*. 2015) and over extensive periods of time (Riegler *et al*. 2005; Weeks *et al*. 2007), limiting the understanding how this bacterium invades new populations.

Horizontal transmission of *Wolbachia* across species boundaries explains the broad distribution of this bacterium. Incongruence between *Wolbachia* and host phylogenies (Baldo *et al*. 2008; Zug *et al*. 2012) and the occurrence of closely related *Wolbachia* strains in unrelated hosts (Baldo *et al*. 2006) are an indirect evidence for the ability of *Wolbachia* to move among species. The evolutionary time spans of *Wolbachia*–host associations are very diverse. For example, the association between bees of the genus *Nomada* and their *Wolbachia* has persisted over 1.7 million years (Gerth *et al*. 2013). An intermediate time span was identified via whole‐genome sequencing of 290 *Drosophila melanogaster* lines and their *Wolbachia*, showing a perfectly congruent phylogeny and suggesting a single ancestral infection event followed by approximately 8000 years of vertical transmission, co‐evolution with its host and some cases of *Wolbachia* loss due to incomplete transmission (Richardson *et al*. 2012). Analysing the *Wolbachia* infection of the North American Eastern cherry fruit fly, *Rhagoletis cingulata*, a recent invader in Europe, showed that *Wolbachia* switched from the European endemic *Rhagoletis cerasi* to *R. cingulata* in Europe in a time frame of <20 years (Schuler *et al*. 2013). Additionally, two other studies on Hymenoptera demonstrated that intraspecific horizontal *Wolbachia* transmission can also play an important role for the spread of *Wolbachia* in new host populations (Kraaijeveld *et al*. 2011; Reumer *et al*. 2012).

Very few examples of ongoing *Wolbachia* invasions into new host populations are documented in field populations (Turelli & Hoffmann 1991; Schuler *et al*. 2013). The dynamics of a newly introduced *Wolbachia* within a host population were studied mainly theoretically by modelling the spread of *Wolbachia* infections due to CI (Turelli *et al*. 1992; Turelli & Hoffmann 1995; Barton & Turelli 2011; Fenton *et al*. 2011; Hancock *et al*. 2011) and tested for a small number of host species in the field, for example *Drosophila simulans* (Kriesner *et al*. 2013) and *Aedes aegypti* (Hoffmann *et al*. 2011). One of the key factors for the successful establishment of a new strain is its ability to induce CI in combination with a high maternal transmission frequency (Hoffmann & Turelli 1997), with possible fecundity advantages of infected over uninfected females (e.g. Fast *et al*. 2011). However, *Wolbachia* can also persist in a population without inducing CI. The examples of persistence of *w*Mel in *D. melanogaster* (Hoffmann *et al*. 1998), *w*Au in *D. simulans* in Australia (Hoffmann *et al*. 1996; Kriesner *et al*. 2013) and *w*Suz in *Drosophila suzukii* in the USA (Hamm *et al*. 2014) show that *Wolbachia* is able to be maintained with minimal or without manipulation of host reproduction. Maternal *Wolbachia* transmission is rarely perfect and can lead to a continuous emergence of uninfected females, hindering fixation of a *Wolbachia* strain (Kriesner *et al*. 2013; Hamm *et al*. 2014). This leakage may, on the other hand, be compensated by selective effects such as CI or beneficial effects of *Wolbachia* provided to its host, including the protection against pathogens (Hedges *et al*. 2008; Fenton *et al*. 2011).

The acquisition of *Wolbachia* can influence the genetic diversity of the maternally transmitted mitochondria (Turelli *et al*. 1992; Hurst & Jiggins 2005); this is of significant importance as mitochondrial DNA (mtDNA) sequences are often used for inferences on species identity, phylogeny and population structure. In populations where infected individuals gain any fitness or reproductive advantage from *Wolbachia*, the mitochondrial genomes of these initially infected individuals will hitchhike with the spreading *Wolbachia*, reducing the haplotype diversity and replacing the haplotypes found in uninfected individuals (Narita *et al*. 2006; Charlat *et al*. 2009; Atyame *et al*. 2011). Therefore, populations recently infected by *Wolbachia* can display a different or fewer mitochondrial lineages than uninfected ones (e.g. Jiggins 2003; Hurst & Jiggins 2005).

**Figure 1:**
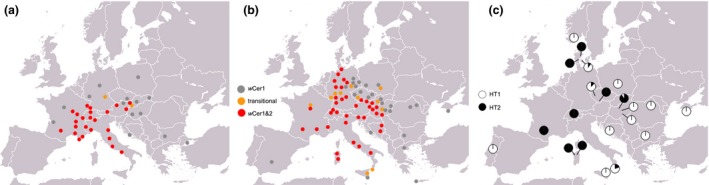
(a) Distribution of unidirectional incompatible southern (red) and northern (grey) *R. cerasi* populations in 1974 with two transitional (orange) populations (Boller *et al*. 1976). (b) Distribution of *w*Cer1 (grey dots), *w*Cer1&2 (red dots) and transitional populations with singly and doubly infected flies (orange dots), modified from Riegler & Stauffer (2002), with inclusion of a subset of representative populations from Fig. 3a,b; (c) prevalence of mitochondrial haplotypes of *R. cerasi* populations in Europe (white HT1, black HT2).

The European cherry fruit fly *R. cerasi* is a model host system to study *Wolbachia* infections in natural populations. It is a serious pest of cherry orchards (Fimiani 1989; Daniel & Grunder 2012). *Rhagoletis cerasi* has a univoltine life cycle and infests cherries, mainly *Prunus avium* and *Prunus cerasus*, and honeysuckle, *Lonicera xylosteum* (Boller & Bush 1974; Schwarz *et al*. 2003). Crossings of males from southern and central European populations with females from northern and eastern European populations showed an egg mortality of 98%, while the reciprocal crosses were fully compatible (Boller *et al*. 1976). Riegler & Stauffer (2002) identified *Wolbachia* as cause of this unidirectional incompatibility. All *R. cerasi* individuals were infected by *w*Cer1, and most central and southern European populations harboured an additional strain, *w*Cer2. In between these two blocks of populations was a transition zone that contained populations with individuals that were either infected by *w*Cer1 or both strains. The geographic distribution of the *w*Cer2 infection closely matched the occurrence of incompatible populations detected by Boller *et al*. (1976) (Riegler & Stauffer 2002; Fig. 1a). A major exception to this pattern was a population 500 km north of the expected transition zone in Germany that was infected by *w*Cer2 in 1998 (Riegler & Stauffer 2002), indicating that *w*Cer2 had either progressed significantly since 1976 or experienced an anthropogenic introduction to northern Germany. Using more sensitive detection techniques, three additional *Wolbachia* strains, *w*Cer3, *w*Cer4, and *w*Cer5, were found at different frequencies in almost all European populations (Arthofer *et al*. 2009b). The prevalence of *w*Cer3 was the lowest and without a clear distribution pattern. The abundance of *w*Cer4 was homogenous across Europe. *w*Cer5 showed differences in spatial distribution not consistent with the distribution of the unidirectional CI phenotype (Arthofer *et al*. 2009b).

Here, we studied the infection dynamics of *Wolbachia*, the distribution of mitochondrial haplotypes and microsatellite allele frequencies in *R. cerasi* in Europe. Furthermore, we focused on the expansion history of *w*Cer2 in *R. cerasi* populations in Germany over a time period of over 15 years and pinpointed the transition zone in which *w*Cer2 introgressed into *w*Cer1‐infected populations (Riegler & Stauffer 2002). Besides establishing a mitochondrial haplotype framework for European populations, we looked at the infections of German field samples from four different collection periods (1998/1999, 2000/2001, 2008 and 2014) in more detail. The previously reported shift in the *Wolbachia* distribution in Germany, between Boller *et al*. (1976) and Riegler & Stauffer (2002), as well as the expected transition zone in central Germany represent an ideal opportunity to study both the spread of the endosymbiont and its influence on the mitochondrial and nuclear genetic structure of *R. cerasi*. A CI‐driven invasion by a new *Wolbachia* strain such as *w*Cer2 was expected to result in a sweep of the infected mitochondrial haplotype and a replacement or reduction of mitochondrial diversity (Turelli *et al*. 1992; Hurst & Jiggins 2005). Thus, a clear association between the spreading *w*Cer2 and a specific mitochondrial *R. cerasi* haplotype would be in support of the expression of CI and reliable maternal inheritance, while a random association would suggest frequent horizontal transmission or loss of *Wolbachia*. Analysis of the nuclear diversity of singly and doubly infected populations would demonstrate whether *Wolbachia* has an impact on the genome of its host. We compared our empirical data with quantitative analyses of the frequency dynamics of *Wolbachia* and associated mitochondrial haplotypes and analysed the invasion front and shifts in the transition zone between the different years.

## Materials and methods

### *Rhagoletis cerasi* collection

Populations of *R. cerasi* were collected from infested *Prunus* (59 populations) and *Lonicera* (33 populations) plants between 1998 and 2014 (Table S1, Supporting information). The fruits were placed in plastic trays at room temperature, and emerging larvae were allowed to pupate and stored in absolute ethanol at −20 °C. DNA extracts of 188 individuals from 20 European populations studied by Riegler & Stauffer (2002) (Fig. 1a, Table S1, Supporting information) were re‐analysed, and one population from Portugal was additionally probed in 2009. Collections from Germany included 103 individuals from 11 populations in 1998/1999 (1‐1 to 1‐11), 226 individuals from 22 populations in 2000/2001 (2‐1 to 2‐22), 468 individuals from 34 populations in 2008 (3‐1 to 3‐34) and 39 individuals from four populations in 2014 (4‐1 to 4‐4; Table S1, Supporting information). Total DNA of single pupae was extracted using a salting‐out method (Miller *et al*. 1988) and dissolved in 50 μL TE buffer.

### Screening for *Wolbachia*

*Wolbachia* screening was performed using *w*Cer1‐ and *w*Cer2‐specific primers, targeting specific regions of the *Wolbachia* surface protein *wsp* (Arthofer *et al*. 2009b). PCR amplification was performed in a total volume of 10 μL using 1× NH_4_ Buffer (Thermo Scientific), 2 mm MgCl_2_, 100 μm dNTPs, 0.2 μm of each primer, 0.25 U *Taq* polymerase (Thermo Scientific) and 0.8 μL template DNA. PCR amplification conditions were 94 °C for 1 min followed by 35 cycles of 94 °C for 30 s, 55 °C for 45 s and 72 °C for 1 min, followed by 72 °C for 10 min. Electrophoretic separation of the PCR products was carried out on 2% ethidium bromide‐stained agarose gels. For the purpose of this study, and in accordance with Riegler & Stauffer (2002), we refer to *w*Cer1‐infected individuals as singly infected, and *w*Cer1&2‐infected individuals as doubly infected. Populations fixed for *w*Cer1 are referred to as singly infected populations, and populations fixed for *w*Cer1&2 as doubly infected populations, while populations with both infection types are transitional populations. Given that an initial screening of the three other *Wolbachia* strains in *R. cerasi* showed a distribution inconsistent with the distribution of CI (Arthofer *et al*. 2009b; data not shown), we did not survey *w*Cer3, *w*Cer4 and *w*Cer5 in this study.

### Mitochondrial genotyping

A 546‐bp fragment of the mitochondrial *COI* gene was amplified using the primers Pat and Dick (Simon *et al*. 1994), and amplicons were Sanger sequenced by a commercial provider. In total, six to 10 individuals from nine European populations outside Germany (Table S1, Supporting information), six individuals from each of seven German populations from 2008 (3‐3, 3‐7, 3‐9, 3‐10, 3‐18, 3‐22, 3‐33), and all 39 individuals from four German populations in 2014 were sequenced. Sequences were aligned using CodonCode Aligner (CodonCode Corporation). All 152 individuals from these 20 populations showed only two haplotypes separated by one polymorphic site. Therefore, in all other individuals (except for the populations from 1998/1999 for which not enough DNA was available) the mitochondrial haplotype was determined by PCR‐RFLP: 0.5 μL of the PCR product was incubated with 0.5 U *Hae*III (Thermo Scientific) at 37 °C for 4 h and loaded on an agarose gel. Haplotype 2 (HT2) was cut into a 342‐ and 204‐bp fragment while haplotype 1 (HT1) remained undigested. DNA extracts of flies with confirmed single and double infections were used as control. Amplicons showing unclear results after PCR‐RFLP were sequenced.

### Nuclear genotyping

Nuclear genotyping was performed on individuals from ten German populations representing different *Wolbachia* infection status, geographically different origins and different host plants (3‐1, 3‐2, 3‐3, 3‐6, 3‐10, 3‐18, 3‐22, 3‐28, 3‐30, 3‐33; Table S1, Supporting information). Seven to 16 individuals per population were genotyped using the seven microsatellite loci RcMic76‐1, RcMic76‐7, RcMic82‐46, RcMic83‐16, RcMic83‐26, RcMic83‐44 and RcMic84‐42 (Arthofer *et al*. 2009a). PCRs were carried out in a total volume of 10 μL containing 1× NH_4_ Buffer, 1.5 mm MgCl_2_, 100 μm dNTPs, 0.2 μm FAM/HEX/NED fluorescent‐labelled M13 primer, 0.02 μm M13 tailed forward primer, 0.2 μm reverse primer, 0.25 U *Taq* polymerase (Thermo Scientific) and 0.8 μL template DNA. Amplification conditions were 94 °C for 5 min followed by 35 cycles at 94 °C for 30 s, 60 °C for 1 min and 72 °C for 45 s with a final extension at 68 °C for 20 min. Fragment separation was performed by capillary electrophoresis on an ABI 3100 sequencer (Applied Biosystems). The electropherograms were visualized with PeakScanner (Applied Biosystems), and alleles were called manually. The overall genetic diversity of the different populations, total number of alleles, number of alleles per population, observed and expected heterozygosity, and deviations from Hardy–Weinberg equilibrium were calculated using genalex ver. 6.5 (Peakall & Smouse 2006), and sequential Bonferroni–Holm corrections (Rice 1989) were performed manually in microsoft excel ^®^. Weir & Cockerham's (1984) *F*_ST_ was calculated separately for the global data set and for each possible pair of populations using fstat ver. 2.9.3.2 (Goudet 1995); the latter approach was chosen as the software only calculates confidence intervals for global, not for pairwise *F*_ST_. The genotype data were then merged to four regional classes (singly infected, population 3‐10; doubly infected north, populations 3‐1, 3‐2, 3‐3; doubly infected south, populations 3‐28, 3‐30, 3‐33; transition zone, populations 3‐6, 3‐18, 3‐22), and this classification was used for amova in genalex. Another amova was computed merging the populations according to their host plant (*Lonicera*, population 3‐18; *Prunus*, all other populations). Nei distances were used as input for a principal coordinate analysis (PCoA) and, together with the geographic distances between populations, for a Mantel test in genalex.

Microsatellite results were used as input for the Bayesian clustering algorithm implemented in structure ver. 2.3.3 (Pritchard *et al*. 2000). The admixture model with correlated allele frequencies was used, with default settings and 120 000 iterations of the Markov chain, discarding the first 20 000 iterations as burn‐in. For each *K* in [1, 10], 10 runs were performed. To identify the best *K*, similarity coefficients and Δ*K* were calculated following the protocol of Evanno *et al*. (2005) as implemented in structuresum ver. 2009 (Ehrich 2006).

## Results

### Distribution of wCer1 and wCer2 across Europe

**Figure 2:**
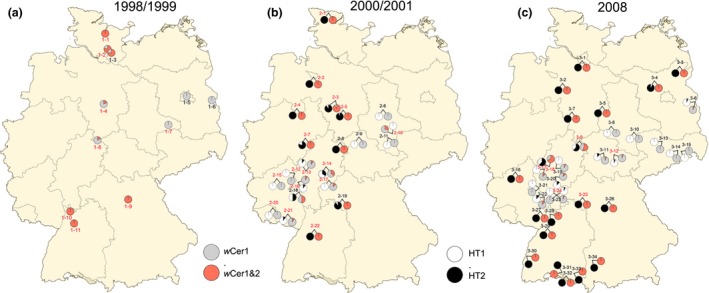
Geographic distribution of *Wolbachia* and mitochondrial haplotypes of *R. cerasi*. (a) *Wolbachia* and mitochondrial haplotype frequencies in 1998/1999, (b) in 2000/2001, and (c) in 2008. White = proportion of individuals from HT1, black = proportion of individuals from HT2, grey = proportion of *w*Cer1 singly infected flies and red = proportion of *w*Cer1&2 doubly infected flies; black numbers represent flies collected from *Prunus* while red numbers represent populations from *Lonicera*. Population localities and numbers are listed in Table S1 (Supporting information).

All 1032 *R. cerasi* individuals analysed in this study were infected with *w*Cer1 (Fig. 1, Table S1, Supporting information). The screening with *w*Cer2‐specific primers confirmed the results of Riegler & Stauffer (2002) that showed fixation of this strain in most parts of southern and central Europe (Fig. 1). Within Germany, our fine‐scale sampling showed fixation of *w*Cer2 in northern and southern Germany (Fig. 2). In contrast, central Germany formed a belt of singly infected populations bordered by transition zones consisting of both singly and doubly infected individuals surrounded by doubly infected populations in the south and the north. These transitional populations contained few to many individuals infected with *w*Cer2 (Fig. 2, Table S1, Supporting information). In contrast to Boller *et al*. (1976) and across the three sampling periods, *w*Cer2 covered all tested populations in Denmark (populations Dan1, Dan2, Dan3), Schleswig‐Holstein (1‐1, 1‐2, 1‐3, 2‐1), Hamburg (3‐1), Bremen (2‐2), Lower Saxony (1‐4, 2‐3, 2‐4, 2‐5, 3‐2, 3‐5, 3‐7, 4‐1, 4‐2), and Witzenhausen in Hesse (1‐8, 2‐8, 3‐9, 4‐3). The portion of *w*Cer2‐infected flies increased in the area around Hamburg (1‐2, 1‐3, 3‐1) and Witzenhausen (1‐8, 2‐8, 3‐9, 4‐3) between 1998 and 2014. Witzenhausen showed differences in the infection status of *R. cerasi* deriving from different host plants: in *Lonicera*‐infesting flies, *w*Cer2 expanded from 14% in 1998 to 43.8% in 2008, while *Prunus‐*infesting flies were already completely invaded by *w*Cer2 in 2000 and 2014.

### Association of *Wolbachia* with mitochondrial haplotypes of *Rhagoletis cerasi*

**Figure 3:**
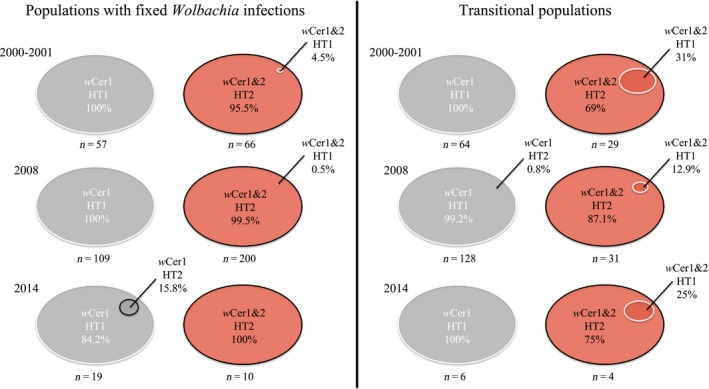
Comparison of the association of *w*Cer1 (grey) and *w*Cer1&2 (red) infections with the two different haplotypes (HT1 white encircled, HT2 black encircled) between populations of Germany outside the transition zone (left) and population from the transition zone (right) collected in 2000/2001, 2008 and 2014. Size of the ovals represents relative abundance of the different *Wolbachia*–haplotype combinations.

The mitochondrial diversity of *R. cerasi* with two detected haplotypes, HT1 (GenBank KJ488948) and HT2 (GenBank KJ488949), was generally very low. Both haplotypes were only separated by a single synonymous transition at a third codon position. Outside Germany, 116 individuals from 15 European populations singly infected with *w*Cer1 were exclusively associated with HT1; in contrast, individuals from doubly infected European populations were almost perfectly associated with HT2, with the exception of one individual from a transitional Danish population on honeysuckle associated with HT1 (Odense; Table S1, Supporting information). This suggests an overall strict association of *Wolbachia* and mitochondrial haplotypes in *R. cerasi* across Europe. In Germany, for which we had more samples and a higher spatial resolution than the rest of Europe, most singly infected individuals were also linked to HT1 and most doubly infected individuals to HT2 (Fig. 3). This association was perfect for all 121 singly infected individuals collected in 2000/2001 that were associated with HT1. In 2008, 236 of 237 *w*Cer1‐infected individuals were associated with HT1, and only one (0.4%) was associated with HT2. In 2014, however, three of 25 *w*Cer1‐infected flies (12%) from a single population (Höhnstedt 4‐4) were associated with HT2.

Outside the transition zones, the strict association of *Wolbachia* and haplotypes was nearly complete: all *w*Cer1 individuals (except Höhnstedt 4‐4) were associated with HT1. The *w*Cer1&2 association with HT2 was also strong, with 63 of 66 (95.5%) individuals in 2000/2001, 199 of 200 (99.5%) individuals in 2008, and 10 of 10 individuals in 2014 (Fig. 3). Within the transition zone, 197 of 198 (99.5%) *w*Cer1 individuals also had HT1, supporting the same pattern as for populations with fixed infection status. The *w*Cer1&2 individuals within the transition zone, however, were different (Fig. 3). In 2000/2001, the association of *w*Cer1&2 with HT2 occurred in only 20 of 29 (69%) individuals, and this was different from populations with fixed infection status (Fisher's exact test, *P *<* *0.001). In 2008, this association occurred in 27 of 31 individuals from the transition zone (87.1%; *P *=* *0.001), and in 2014 in three of four individuals from the transition zone (75%; *P *=* *0.029) (Fig. 3). In total, 21.9% (14 of the 64) *w*Cer1&2 individuals from the transition zone were associated with HT1. This is in sharp contrast to populations outside the transition zone where just four of 276 (1.5%; *P *<* *0.001) doubly infected individuals were associated with HT1. Additionally, the haplotype association of doubly infected flies differed between host plants: while 12 of 47 (25.5%) *w*Cer1&2 flies from *Lonicera* were associated with HT1, just two of 17 (11.8%; *P *=* *0.53) *w*Cer1&2 flies from *Prunus* showed this association.

### No association of *Wolbachia* with nuclear genome of *Rhagoletis cerasi*

Of 116 individuals screened at seven nuclear microsatellite loci, 89.7% of the reactions amplified successfully. Eleven locus–population pairs showed significant deviation in the chi‐square tests for Hardy–Weinberg equilibrium, and one pair retained significance after sequential Bonferroni–Holm correction (Table S2, Supporting information). Both global *F*_ST_ = 0.019 (Table S3, Supporting information) and population pairwise *F*_ST_ (min. −0.032, max. 0.076, Table S4, Supporting information) were low, and, with two exceptions (population pairs 3‐10/3‐18, 3‐18/3‐30), the confidence interval for *F*_ST_ included zero. Infection‐based amova allocated 0% variation among the infection classes (singly infected, doubly infected north, doubly infected south, transition zone), 3% among populations, 20% among individuals and 77% within individuals, respectively (Table S3, Supporting information). The Mantel test (*R* = −0.053, *P* = 0.409) rejected geographic structuring of the genotypes. Host plant‐based amova allocated 3% variation among the host plant classes (*Lonicera* and *Prunus*), 1% among populations, 20% among individuals and 76% within individuals (Table S5, Supporting information). The first three axes of the PCoA explained cumulatively 68.25% of the total variation, and no obvious clusters were observed (Table S6, Supporting information). Evanno analysis of the structure data resulted in a maximum of Δ*K* at *K* = 2. This method is unable to identify *K* = 1 as best estimation of *K*, but visual inspection of the structure box plots and the distribution of Ln P(D) did not show population structuring (Fig. S1, Supporting information). Summarizing, several distance‐, frequency‐ and Bayesian inference‐based analysis methods applied on this data set agreed that there was no pronounced nuclear population structure in Germany.

### Mathematical modelling and quantitative analysis

The data set was analysed quantitatively using mathematical models (see Appendix I for detailed model descriptions). First, we followed Hoffmann *et al*. (1990) and described the spread of CI‐inducing *Wolbachia* in a panmictic population by a nonlinear recursion equation. Three parameters were included: the level of cytoplasmic incompatibility (*l*_CI_), the maternal transmission rate (1 − µ) and the relative fecundity of infected females in comparison with uninfected females (*F*). In order to simulate the spread of *w*Cer2 in a *w*Cer1 population, we had to estimate parameter values. Based on crossing studies by Boller *et al*. (1976), we assumed unidirectional CI with a CI level of 0.98 between doubly (*w*Cer1&2) and singly (*w*Cer1) infected individuals. For transmission, we assumed 100% maternal transmission. This is because field data of *R. cerasi* indicated high infection prevalence for *w*Cer2 and a low number of singly infected individuals with HT2. As we had no information about potential fitness costs of the *Wolbachia* infection in *Rhagoletis*, we assumed no fecundity reduction due to *Wolbachia*.

Next, we investigated the spatial spread of *w*Cer2 in Europe. In general, CI‐inducing *Wolbachia* are predicted to spread spatially as a travelling wave (Turelli & Hoffmann 1991; Schofield 2002, see discussion for alternative model approaches). Let σ be the variance of the individual dispersal probability. Under the assumption of a Gaussian dispersal kernel, 100% maternal transmission rate and no fecundity cost of infection (*F* = 1), the width of the transition zone (defined as the geographic range in which *Wolbachia* frequency increases from 5% to 95%) is predicted as $\Delta x=3\sigma /\sqrt{l_{\mathrm{CI}}}$ (Turelli & Hoffmann 1991). This theoretical prediction was compared with the empirical data. To obtain a good estimate of the transition zone's width and because sampling coverage was highest for this region, we focused on the situation in Germany. First, we noted that the spread of *w*Cer2 most likely happened along a north–south axis (Fig. 2). Therefore, a lower bound for the transition zone width Δ*x* was given by the latitudinal distance between the most northern and the most southern populations that contained both singly and doubly infected individuals. For 1999, the latitudinal distance between Ahrensburg (1‐2) and Witzenhausen (1‐8) was 260 km, which was set as a lower bound for the transition zone width. For 2008, the latitudinal distance between Witzenhausen (3‐9) and Stockstadt (3‐22) was 170 km. However, *w*Cer2 spread from both north and south into the transition zone. Therefore, a lower bound of Δ*x* was given by half of this distance, that is Δ*x* > 130 km for 1999 and Δ*x *>* *85 km for 2009. These two lower bounds were then used to estimate minimal values of σ. Using equation $\Delta x=3\sigma /\sqrt{l_{\mathrm{CI}}}$ for a CI level of 0.98 yields lower estimates for the dispersal kernel of σ > 43 km for 1999/2000 and of σ > 28 km for 2008. However, adult cherry flies are estimated to fly a maximum distance of 4 km (Boller & Remund 1983). This suggests that long‐distance migration of adults or dispersal of infested fruits is key for understanding the spatial spread of *w*Cer2.

**Figure 4:**
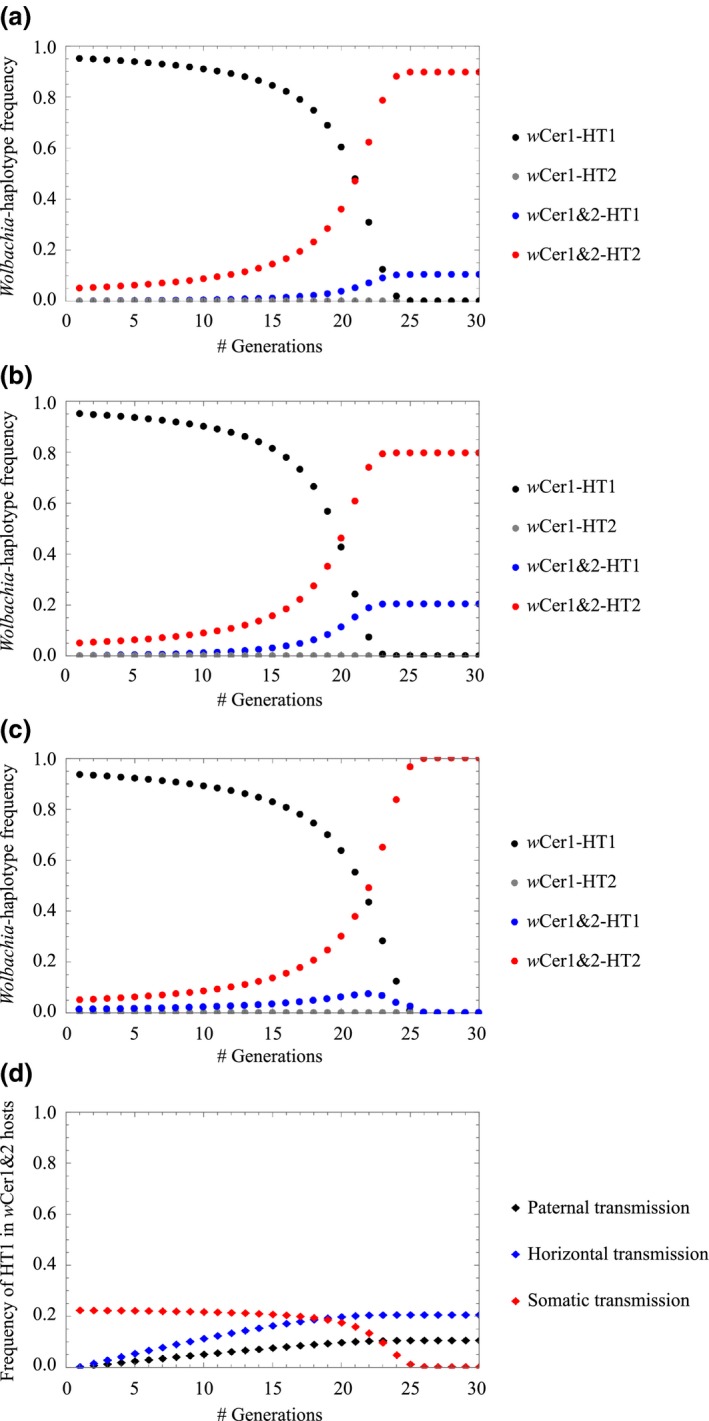
Codynamics of *Wolbachia* and mitochondrial haplotypes. (a) Model with paternal transmission (b) model with heritable horizontal transmission, (c) model with somatic horizontal transmission, (d) dynamics of *Wolbachia*–haplotype mismatch for all three models. *Wolbachia*–haplotype combination frequencies were determined using equations (A8), (A9), (A10), (A11), (A12), (A13), (A14), (A15), (A16), (A17), (A18), (A19). Parameters: *l*_CI_ = 0.98, µ = 0, *F*_*A*_ = *F*_*B*_ = 1 for all graphs; τ = 0.3, β = 0 for model with paternal transmission; τ = 0, α = 1, β = 0.015 for model with horizontal transmission; τ = 0, α = 0, β = 0.3 for model with somatic transmission.

Some doubly infected individuals in the transition zone have haplotype HT1 (21.8%; Fig. 3), but this combination was uncommon outside the transition zone. This high incidence of unexpected *Wolbachia*–haplotype combination could be the result of either paternal (e.g. Hoffmann & Turelli 1988) or intraspecific horizontal transmission of *Wolbachia*. To examine this further, we analysed three extensions of the basic CI model: (i) paternal transmission, (ii) horizontal transmission with subsequent vertical transmission (heritable horizontal transmission) and (iii) somatic horizontal transmission with no vertical transmission (transient horizontal transmission) (see Appendix I for details). Within this theoretical framework, we analysed the population dynamics of *Wolbachia* and the mitochondrial haplotypes. As expected, the *Wolbachia*–haplotype combination *w*Cer1&2‐HT1 was formed in all three models (Fig. 4a–c). Differences between the three models occurred with respect to the long‐term dynamics. The mismatched combination continued to persist in the model with paternal transmission (Fig. 4a) and heritable horizontal transmission (Fig. 4b), but went to extinction in the model with somatic horizontal transmission (Fig. 4c). In conclusion, all three models could explain the data within the transition zone, but only the model with somatic horizontal transmission could explain the absence of the mismatched *Wolbachia*–haplotype combination *w*Cer1&2‐HT1 outside the transition zone (Fig. 4d).

## Discussion

We investigated the dynamics of the *Wolbachia* strain *w*Cer2 and its co‐inheritance with haplotype HT2 in *R. cerasi* in Europe over a time period of over 15 years. A special focus was on central Germany, where *w*Cer2 was previously reported to spread into *w*Cer1 singly infected populations (Riegler & Stauffer 2002). Our theoretical analysis of the transition zone based on distributional shifts within this time frame shows a large transition zone of *w*Cer2, which may be driven by long‐distance migration of *R. cerasi*. We further investigated mitochondrial diversity of the German *R. cerasi* populations and found a strong association of the infection status of *w*Cer1 and *w*Cer1&2 with the two mitochondrial haplotypes HT1 and HT2, respectively. However, in transitional populations we detected 21.8% of *w*Cer2‐infected individuals with HT1, suggesting a high degree of intraspecific somatic horizontal transmission. Mathematical modelling in combination with the evidence for the strong *Wolbachia–*haplotype associations in populations outside the transition zone suggests that the horizontal acquisition of *w*Cer2 detected in the transition zone is most likely transient. Finally, we found no impact of the *w*Cer2 spread on nuclear diversity of its host.

### A rapid spread of wCer2

The *Wolbachia* strain *w*Cer1 was fixed in all populations confirming previous findings (Riegler & Stauffer 2002; Arthofer *et al*. 2009b). Riegler & Stauffer (2002) found mostly geographic congruence between the distribution of mating incompatibilities described by Boller *et al*. (1976) and the occurrence of *w*Cer1 and *w*Cer1&2 after 26 years (1976–2002). However, this previous study also demonstrated that populations in Austria with previously reported incompatibilities (Boller *et al*. 1976) have become completely invaded by *w*Cer2 (Fig. 1). Further comparison of the sampling locations of incompatible populations in the 1970s and the occurrence of *w*Cer2 in the late 1990s established that the range of *w*Cer2 has expanded further from Austria to Western Hungary, and it was also detected in Bosnia‐Herzegovina (Riegler & Stauffer 2002; Fig. 1). While the incompatibility caused by *w*Cer2 originally seemed to be restricted to the southern and central European populations, Riegler & Stauffer (2002) described a population in northern Germany (Kiel) infected by *w*Cer1&2. Here, we further confirm this by describing *w*Cer2 in three Danish populations and by our fine‐scale screening of populations of the German transition area in 1998/1999, 2000/2001, 2008 and 2014. It is not entirely clear how *w*Cer2 expanded into populations of Denmark and northern Germany. The accidental release of *w*Cer1&2 individuals from southern populations due to the transport of infested cherries to the north could be the origin of the double infections in the north.

An interesting finding is the large width of the transition zone with lower estimates of 260 km for 1999 and 170 km for 2008. Under the assumption of a Gaussian dispersal kernel, the quantitative analysis yields lower estimates of the average dispersal probability of 43 km for 1999 and 28 km for 2008. These lower bounds are in stark contrast to empirical studies of *R*. *cerasi* that estimate maximal migration distances of single individuals around 4 km (Boller & Remund 1983), suggesting that long‐distance migration or passive movement of flies plays an important role. The latter could be possibly facilitated by human transport of infested cherry fruits. It could also be possible that previous mark/recapture studies (Boller & Remund 1983) underestimated the migration of *R. cerasi*, although a migration of adults more than 20 km seems unlikely.

Studies of *Wolbachia* in natural host populations showed that this endosymbiont can experience different patterns of range expansions in the field: field released *A. aegypti* mosquitos that have been artificially infected with *w*Mel (Hoffmann *et al*. 2011) suffer small but significant fitness costs that can hinder the spread of *Wolbachia* outside the released areas (Hoffmann *et al*. 2014). This was tested, and low‐frequency introduction into surrounding areas did not result in *Wolbachia* establishment, suggesting structured host populations and bistable dynamics with unstable equilibrium frequencies. In contrast, the spatial spread of *w*Ri in *D. simulans* in California (Turelli & Hoffmann 1991) and Australia may follow a Fisherian wave (Kriesner *et al*. 2013). For this system, it was shown that *w*Ri increases female fecundity, resulting in a spread from low initial infection frequencies. Kriesner *et al*. (2013) argued that this promotes the spatial spread of *Wolbachia* because rare long‐distance migration can result in the establishment of *Wolbachia*‐infected satellite populations. This is different from situations with bistable dynamics where long‐distance migration has no significant effect on spatial spread because low *Wolbachia* infections are quickly lost. For the quantitative analysis of our data, we chose the ‘standard model’ of Barton & Turelli (2011) because we do not have data about potential fecundity effects of *w*Cer2. Without available data about potential fitness benefits, an important factor for *Wolbachia* spread from initial low frequencies (Fenton *et al*. 2011; Kriesner *et al*. 2013), alternative approaches that evaluate the spread based on Fisherian dynamics (Kriesner *et al*. 2013) are not suitable for our data. Future studies are necessary to characterize the phenotypic effects of *w*Cer2 on its host (other than CI) in conjunction with continued monitoring in a number of sites over a series of years. All of these efforts will be needed to analyse the spread of *w*Cer2 in *R. cerasi* more comprehensively.

### Low mitochondrial diversity and the association of specific haplotypes with *Wolbachia*

Characterizing the mtDNA of 733 *R. cerasi* individuals from different populations in Germany and 196 individuals from other European populations revealed only two closely related haplotypes. This remarkably low mitochondrial diversity in widely distributed populations of *R. cerasi* is therefore best explained by two consecutive sweeps, first of *w*Cer1 and then of *w*Cer1&2. This first invasion by *w*Cer1 is expected to have begun from a single or very few founder individuals that had acquired this strain horizontally from another species. Subsequent fitness and/or reproductive advantages of *w*Cer1‐infected individuals, for example due to the induction of CI, resulted in the spread of *w*Cer1 and the elimination of uninfected haplotypes. Due to the fact that *w*Cer1 reached fixation in all *R. cerasi* populations, a comparison of mtDNA diversity between infected and uninfected populations (e.g. Atyame *et al*. 2011) is not possible for *R. cerasi*.

The origin of HT2 remains an unresolved issue, with two possible scenarios. First (and perhaps most likely), invasion of *w*Cer1 had occurred long enough prior to the invasion of *w*Cer2 to allow the evolution of new mitochondrial diversity within *w*Cer1‐infected populations. Second, *w*Cer1 invaded independently two different and uninfected *R. cerasi* haplotypes HT1 and HT2, resulting in the extinction of all but these two haplotypes. HT2 later acquired *w*Cer2 and since then has hitchhiked with *w*Cer2 through European populations. Both scenarios require that HT2, when acquiring *w*Cer2, was a spatially isolated haplotype, as otherwise singly infected HT2 populations should have been detected; furthermore, the spread of *w*Cer2 and HT2 must have been fairly recent. Current data do not discriminate between the two scenarios, rendering a determination of the age of the spread based on molecular clock calculations (e.g. Rasgon *et al*. 2006) impossible. However, both hypotheses indicate that the spread of both *w*Cer1 and *w*Cer2 started from just a few, if not one single individual, thus driving the mitochondrial genome of *R. cerasi* through a severe genetic bottleneck.

### Nearly perfect maternal transmission

Because both mitochondria and *Wolbachia* are cytoplasmically inherited, their association provides information about the efficiency of the *Wolbachia* spread. The detection of only four of 415 genotyped individuals (0.96%) with mitochondrial HT2 not being infected by *w*Cer2 suggests that this strain induces very strong CI and probably has high transmission efficiency. We assume that rare associations of *w*Cer1 singly infected individuals with HT2 seen in the field are due to imperfect transmission of *w*Cer2, and we also assume that this haplotype–*Wolbachia* strain association is transient: in case of a male individual, the association is already in a dead end; singly infected females, on the other hand, may likely fail in finding a compatible mate. Of HT2 individuals from within the transition zone only 1.96% (one of 51) were not infected by *w*Cer2. This further supports the view of strong CI induction capacity and high transmission efficiency of *w*Cer2. Our theoretical predictions are supported by studies of Boller *et al*. (1976) that showed nearly complete cytoplasmic incompatibility of 98% (Riegler & Stauffer 2002).

### Frequent intraspecific transient horizontal transmission

An unexpected finding, however, was the presence of *w*Cer2 in HT1 individuals (Fig. 3). Considering just populations from the transition zone, 21.9% of the *w*Cer2‐infected individuals were associated with HT1. We cautiously interpret this observation as evidence for repeated intraspecific horizontal transmission events of *w*Cer2 into singly infected HT1 flies, without transmission into the next generation. Although horizontal transmission of *Wolbachia* is commonly found on an evolutionary timescale (O'Neill *et al*. 1992; Vavre *et al*. 1999; Baldo *et al*. 2008; Kraaijeveld *et al*. 2011; Gerth *et al*. 2013) and rarely observed in real time in the field (Schuler *et al*. 2013), it does not necessarily lead to a successful establishment in new populations. In line with our observations, recent studies on Australian tephritid fruit flies sharing host plants and parasitoids demonstrated that identical *Wolbachia* was detected across fruit fly and parasitoid species (Morrow *et al*. 2014), however at an overall low prevalence within most species (Morrow *et al*. 2015). This suggests that *Wolbachia* can readily move between closely interacting species even if prevalence and maternal transmission is low; yet, this *Wolbachia* spillover may be transient and not passed on to the next generation (Morrow *et al*. 2015). Thus, a contribution of parasitoids to the transmission of *w*Cer2 into HT1 seems plausible under the assumption that at least some flies survive the parasitoid attack. It should be noted, however, that none of the mtDNA sequenced individuals gave any hints for the presence of parasitoid DNA. Another route for intraspecific horizontal transmission could be cannibalism by differently infected larvae that co‐inhabit the same host fruits.

An alternative explanation for the findings of *w*Cer2 in HT1 individuals could be paternal transmission of *Wolbachia* (e.g. Hoffmann & Turelli 1988). The strong CI caused by *w*Cer2 minimizes the number of progeny from *w*Cer2‐HT2 male and *w*Cer1‐HT1 female crosses and thus the likelihood that offspring inherits HT1 from the mother and *w*Cer2 from the father. This is supported by theoretical predictions showing that in case of paternal *w*Cer2 transmission more than 10% of the individuals would be permanently associated with HT1 (Fig. 4c).

The question remains whether transferred *w*Cer2 ever reaches the germline of HT1 flies (and is eventually inherited) or remains a somatic infection. Successful and permanent invasion of the germline would result in a permanent association of 20% *w*Cer2‐infected individuals with HT1 (Fig. 4a); however, this was in discordance with our empirical data that found *w*Cer1&2‐HT1 individuals almost exclusively in the transition zone (Fig. 2). Such findings of potentially transient, not inherited somatic infections, are further evidenced by laboratory studies demonstrating that *Wolbachia*, even if successfully transferred by microinjection, can be lost in a few generations due to insufficient maternal transmission (e.g. Riegler *et al*. 2004). We therefore assume that in our case infections acquired by intraspecific horizontal transmission either do not invade the host's germline or suffer from poor maternal transmission (Riegler *et al*. 2004). Theoretical analyses of our data support this scenario and demonstrate that temporarily more than 20% of the individuals can show the *w*Cer1&2–HT1 association that will be lost after complete invasion of (maternally transmitted) *w*Cer2 (Fig. 4c).

### A potential role of the host plant in the wCer2 invasion

While within the transition zone, 25.5% *w*Cer1&2‐infected flies from *Lonicera* were associated with HT1, just 11.8% *w*Cer1&2‐infected flies from *Prunus* were associated with HT1, suggesting a potential role of the host plant in horizontal transmission. Smaller size of *Lonicera* berries may increase the likelihood of cannibalism between larvae that share a fruit resulting in horizontal transmission of *Wolbachia*. Alternatively, if indeed parasitoids are involved in horizontal *Wolbachia* transmission (Gehrer & Vorburger 2012; Ahmed *et al*. 2015), adult parasitoids emerging from the earlier occurring cherry host could provide high *w*Cer2 loads to the later attacked honeysuckle‐infesting larvae. In contrast, the first emerging parasitoids of each year, attacking the cherry host, would not yet have had a chance to acquire *w*Cer2.

An interesting case was found in the northern transition zone at Witzenhausen (populations 1‐8, 2‐8, 3‐9, 4‐3). The samples from 1999 to 2008 were collected from honeysuckle, while in 2000 and 2014 cherry had been sampled, and the results revealed another possible host plant effect: On honeysuckle, *w*Cer2 prevalence increased from 17% in 1999 to 56% in 2008, while on cherry *w*Cer2 had reached fixation already in 2000 (and stayed fixed in 2014). Such a delayed increase on honeysuckle could indicate that *w*Cer2 faces a number of challenges when invading populations of *R. cerasi*. The potential for host race formation of *R. cerasi* on cherry and honeysuckle has previously been discussed (Boller *et al*. 1998; Schwarz *et al*. 2003) and could impede a *Wolbachia* invasion due to host plant phenology, with cherries becoming available prior to honeysuckle berries and female host plant preference being determined by previous female experience (Boller *et al*. 1998; but see results of our microsatellite analysis). Furthermore, *R. cerasi* is a univoltine insect with an obligatory diapause and a portion of pupae undergoing prolonged dormancy. These overlaying pupae emerge in the subsequent year(s) (Vallo *et al*. 1976; Moraiti *et al*. 2014) and could thus act as reservoir of singly infected flies that delay *w*Cer2 fixation.

To further investigate the role of the *w*Cer2 spread on its host, we characterized the nuclear diversity of singly and doubly infected *R. cerasi* populations using previously developed microsatellite loci (Arthofer *et al*. 2009b). Characterization of different populations of *R. cerasi* showed that neither the different *Wolbachia* infection, nor geographic separation resulted in any nuclear genetic structure of *R. cerasi*. Furthermore, we could not detect genetic differences in sympatrically overlapping *Prunus‐* and *Lonicera*‐infesting host forms, concluding that *w*Cer2 did not sufficiently inhibit gene flow to result in population divergence. This is in line with a previous study that showed that unlike large effects on mtDNA the spread of *Wolbachia* has little effects on nuclear genomes (Turelli *et al*. 1992). However, our observation of at least some differences at the *Wolbachia* invasion front between *Prunus* and *Lonicera* might be an additional indication (besides data presented by Boller *et al*. 1998; Schwarz *et al*. 2003) of the formation of host races in *R. cerasi* and merits further investigation.

## Conclusion

We studied the infection dynamics of *Wolbachia* in *R. cerasi* in Europe and focussed on the invasion history of *w*Cer2 over a time period of 15 years. The comparison of our data with studies from the 1970s and our fine‐scale analysis of populations from Germany show that *w*Cer2 is currently invading *w*Cer1‐infected populations from the south and from the north. Our quantitative analysis yielded a large transition zone of *w*Cer2 that suggests a spreading *w*Cer2 infection in Germany. Furthermore, we show low mitochondrial diversity and a high level of mitochondrial haplotype association in this host species. *w*Cer1 singly infected populations are almost perfectly associated with HT1 and populations with fixed *w*Cer2 infections perfectly associated with HT2. The transitional zone, remarkably, showed a large proportion of HT1 flies infected by *w*Cer2, suggesting a high frequency of intraspecific horizontal transmission. However, as the *w*Cer2–HT1 association appears to be almost exclusive to the transition zone, we assume that this combination is due to horizontal transmission of *w*Cer2 that seems to be transient. Theoretical modelling supports this assumption. The rare reciprocal *w*Cer1–HT2 combination suggests nearly perfect maternal transmission of *w*Cer2. In summary, our study constitutes a new example of a *Wolbachia* spread in natural populations and provides novel insights into the dynamics of natural *Wolbachia* invasion in the field.

The project was conceived and designed by M.R., C.S., W.A. and H.S. The data collection and analysis were performed by H.S., M.R., W.A., S.D.H., K.K., B.R. and S.K. A.T. designed the mathematical model and conducted the quantitative analysis. Materials and specimens were supplied by M.R., K.K., D.S., T.H. and C.S. The manuscript was written by H.S., A.T., C.S., W.A. and M.R. with contributions from all other authors.

## Data accessibility

Mitochondrial DNA sequences have been submitted to GenBank: Accession numbers: KJ488948 (HT1) and KJ488949 (HT2). Mitochondrial DNA sequence chromatograms, microsatellite raw data, genalex, fstat, and structure data and result files have been deposited at Dryad: Provisional DOI: 10.5061/dryad.gs8r.2.

## Supporting information


**Fig. S1** Results of structure analysis of microsatellite data.

**Table S1** Locality information, frequency of *Wolbachia* infections (*w*Cer1, *w*Cer1&2), frequencies of different haplotypes, and association of *Wolbachia* with the respective haplotype.
**Table S2** Summary of microsatellite data chi‐square tests for Hardy–Weinberg Equilibrium.
**Table S3** Results from amova analysis based on infection status.
**Table S4** Results from pairwise *F*_ST_ analysis.
**Table S5** Results from amova analysis based in *R. cerasi* host plants.
**Table S6** Principal coordinate analysis (PCoA) of different *R. cerasi* populations, based on Nei distances.
**Table S7** Mating table used to derive model (A4), (A5), (A6), (A7). Modified from Turelli *et al*. (1992).

## Appendix I Mathematical modelling and quantitative analysis

### Infection dynamics of CI‐inducing *Wolbachia*

#### CI dynamics in a panmictic population

We followed Hoffmann *et al*. (1990) and describe the infection dynamics of CI‐inducing *Wolbachia* in a panmictic host population by a nonlinear recursion equation. Host generations are discrete and nonoverlapping. Let *p*_*w*_ and *p*′_*w*_ denote the frequency of *Wolbachia*‐infected hosts in subsequent generations. Then, the intergenerational change in infection frequency is described by

(A1) $$p_{w}^{\prime }=\frac{F\left(1-\mu \right)p_{w}}{1-l_{\mathrm{CI}}p_{w}\left(1-p_{w}\right)-\mu l_{\mathrm{CI}}p_{w}^{2}},$$

where *F* denotes the relative fecundity of infected females in comparison with uninfected ones, (1 − μ) the maternal transmission rate of *Wolbachia*, and *l*_CI_ the level of cytoplasmic incompatibility, defined as offspring loss in incompatibility matings (i.e. infected male and uninfected female) in comparison with the other possible matings. Note that model (A1) does not allow for paternal or horizontal transmission of *Wolbachia*.

#### Spatiotemporal model

The spatiotemporal spread of CI‐inducing *Wolbachia* was previously modelled using partial differential equations (Turelli & Hoffmann 1991; Schofield 2002). Let *p* = *p* (*x, t*) denote the frequency of *Wolbachia* at point x∈R and time t∈R. Under the basic assumptions of model (A1) with respect to transmission, fecundity reduction and CI, the spatial dynamics of *Wolbachia* is described by

(A2) $$\frac{\partial p(x,t)}{\partial t}=\frac{\sigma^{2}}{2}\frac{\partial^{2}p\left(x,t\right)}{\partial x^{2}}+l_{\mathrm{CI}}p\left(x,t\right)\left(1-p\left(x,t\right)\right)\left(p\left(x,t\right)-p*\right)-\mu Fp\left(x,t\right),$$

where $p^{*}=1-F/l_{\mathrm{CI}}$ is the instable fix point of model (A1) for µ = 0 and σ is the variance of the individual movement probability distribution of host individuals. Analytical solutions exist for µ = 0. For *p** < 0.5, *Wolbachia* is expected to spread as a travelling wave,

(A3) $$p\left(x,t\right)=\frac{1}{2}\left[1+\tanh\left(\frac{x+vt}{2w}\right)\right],$$

where $v=\frac{1}{2}\left(1-p^{*}\right)\sqrt{l_{\mathrm{CI}}}\sigma$ is the wave speed and $w=\sigma /\sqrt{l_{\mathrm{CI}}}$ the wave width, such that the *Wolbachia* frequency is expected to go from *p* ≈ 0.5 to *p* ≈ 0.9 over Δ*x* = 3*w* (Turelli & Hoffmann 1991). We refer to Δ*x* as to the transition zone width.

### Codynamics of CI‐inducing *Wolbachia* and mitochondrial haplotypes

#### Model with paternal transmission

Turelli *et al*. (1992) analysed an extension of model (A1) to investigate the effect of paternal transmission of *Wolbachia* on the codynamics of *Wolbachia* and mitochondrial haplotypes. There are two mitochondrial haplotypes (*M*_1_ and *M*_2_), and individuals can be either infected with *Wolbachia* (*W*) or not (Ø). The model describes the intergenerational change in frequency of the four *Wolbachia*–haplotype combinations (denoted by *p*_*W*1_, *p*_*W*2_, *p*_01_ *p*_02_). It is derived using table S7 and computes to

(A4) $$\bar{W}p_{w1}^{\prime }=F_{1}\left(1-\mu \right)p_{w1}+\tau H\left(F_{1}\mu p_{w1}+p_{Ø1}\right)\left(p_{w1}+p_{w2}\right),$$

(A5) $$\bar{W}p_{w2}^{\prime }=F_{2}\left(1-\mu \right)p_{w2}+\tau H\left(F_{1}\mu p_{w2}+p_{Ø2}\right)\left(p_{w1}+p_{w2}\right),$$

(A6) $$\bar{W}p_{Ø1}^{\prime }=\left(p_{Ø1}+F_{1}\mu p_{w1}\right)\left[p_{Ø1}+p_{Ø2}+\left(1-\tau \right)H\left(p_{w1}+p_{w2}\right)\right],$$

(A7) $$\bar{W}p_{Ø2}^{\prime }=\left(p_{Ø2}+F_{2}\mu p_{w2}\right)\left[p_{Ø1}+p_{Ø2}+\left(1-\tau \right)H\left(p_{w1}+p_{w2}\right)\right],$$

where (1 − μ) and τ are the maternal and the paternal transmission rates of *Wolbachia*, respectively, *H* = 1 − *l*_CI_ the relative hatch rate in incompatibility matings, *F*_*i*_ the relative fecundity of *Wolbachia*‐infected females with haplotype *M*_*i*_ relative to uninfected females, and $\overset{¯}{W}$ the average fitness defined as the sum of all terms on the right‐hand side of equations (A4), (A5), (A6), (A7). The original model analysed by Turelli *et al*. (1992) is the special case of (A4), (A5), (A6), (A7) for *F*_1_ = *F*_2_.

#### Model with horizontal transmission

We extended model (A4), (A5), (A6), (A7) to incorporate horizontal transmission of *Wolbachia*. Horizontal transmission follows the mass action principle and is described by parameter β. All horizontally transmitted *Wolbachia* establish a somatic infection in the new host, but only the fraction α of these enters the germ line and is inherited to future generations. Somatic infections are not further transmitted and a dead end for *Wolbachia*. Horizontally transmitted *Wolbachia* can neither induce CI nor protect against it.

Let $\left(p_{w1},p_{w2},p_{Ø1},p_{Ø2}^{\prime }\right)\;\;\text{and}\;\;\left(p_{w1}^{\prime },p_{w2}^{\prime },p_{Ø1}^{\prime },p_{Ø2}^{\prime }\right)$ denote the *Wolbachia*–haplotype frequencies in the germline in subsequent generations among adults during mating. The change in frequency is described as follows. First, we calculate the frequencies among offspring, denoted by $\left(p_{w1}^{+},p_{w2}^{+},p_{Ø1}^{+},p_{Ø2}^{+}\right)$. These compute to

(A8) $$\bar{W}p_{w1}^{+}=F_{1}\left(1-\mu \right)p_{w1}+\tau H\left(F_{1}\mu p_{w1}+p_{Ø1}\right)\left(p_{w1}+p_{w2}\right),$$

(A9) $$\bar{W}p_{w2}^{+}=F_{2}\left(1-\mu \right)p_{w2}+\tau H\left(F_{1}\mu p_{w2}+p_{Ø2}\right)\left(p_{w1}^{+}p_{w2}\right),$$

(A10) $$\bar{W}p_{Ø1}^{+}=\left(p_{Ø1}+F_{1}\mu p_{w1}\right)[p_{Ø1}+p_{Ø2}+\left(1-\tau \right)H\left(p_{w1}+p_{w2}\right)],$$

(A11) $$\bar{W}p_{Ø2}^{+}=\left(p_{Ø2}+F_{2}\mu p_{w2}\right)[p_{Ø1}+p_{Ø2}+\left(1-\tau \right)H\left(p_{w1}+p_{w2}\right)],$$

where all parameters and $\overset{¯}{W}$ are defined as in model (A4), (A5), (A6), (A7).

Next, horizontal transmission takes place. We first consider only somatic infections of *Wolbachia*. Let $\left({\tilde{p}}_{w1},{\tilde{p}}_{w2},{\tilde{p}}_{Ø1},{\tilde{p}}_{Ø2}\right)$ denote the *Wolbachia*–haplotype frequencies for host individuals that are infected at germline and/or soma. Then, it holds that

(A12) $${\tilde{p}}_{w1}=p_{w1}^{+}+\beta p_{Ø1}^{+}\left(p_{w1}^{+}+p_{w2}^{+}\right),$$

(A13) $${\tilde{p}}_{w2}=p_{w2}^{+}+\beta p_{Ø2}^{+}\left(p_{w1}^{+}+p_{w2}^{+}\right),$$

(A14) $${\tilde{p}}_{Ø1}=p_{Ø1}^{+}-\beta p_{Ø1}^{+}\left(p_{w1}^{+}+p_{w2}^{+}\right),$$

(A15) $${\tilde{p}}_{Ø2}=p_{Ø2}^{+}-\beta p_{Ø2}^{+}\left(p_{w1}^{+}+p_{w2}^{+}\right),$$

where 0 ≤ β ≤ 1.

The fraction α of somatic infections enters the germline. Accordingly, the *Wolbachia*–haplotype frequencies in the next generation compute to

(A16) $$p_{w1}^{\prime }=p_{w1}^{+}+\alpha \beta p_{Ø1}^{+}\left(p_{w1}^{+}+p_{w2}^{+}\right),$$

(A17) $$p_{w2}^{\prime }=p_{w2}^{+}+\alpha \beta p_{Ø2}^{+}\left(p_{w1}^{+}+p_{w2}^{+}\right),$$

(A18) $$p_{Ø1}^{\prime }=p_{Ø1}^{+}-\alpha \beta p_{Ø1}^{+}\left(p_{w1}^{+}+p_{w2}^{+}\right),$$

(A19) $$p_{Ø2}^{\prime }=p_{Ø2}^{+}-\alpha \beta p_{Ø2}^{+}\left(p_{w1}^{+}+p_{w2}^{+}\right),$$

where 0 ≤ α ≤ 1.
