# Supplementary figures and images for: The hitchhiker's guide to Europe: the infection dynamics of an ongoing Wolbachia invasion and mitochondrial selective sweep in Rhagoletis cerasi
Source: Mol Ecol. 2016 Mar 15;25(7):1595–609. doi: 10.1111/mec.13571 (PMC4950298; doi:10.1111/mec.13571)

A

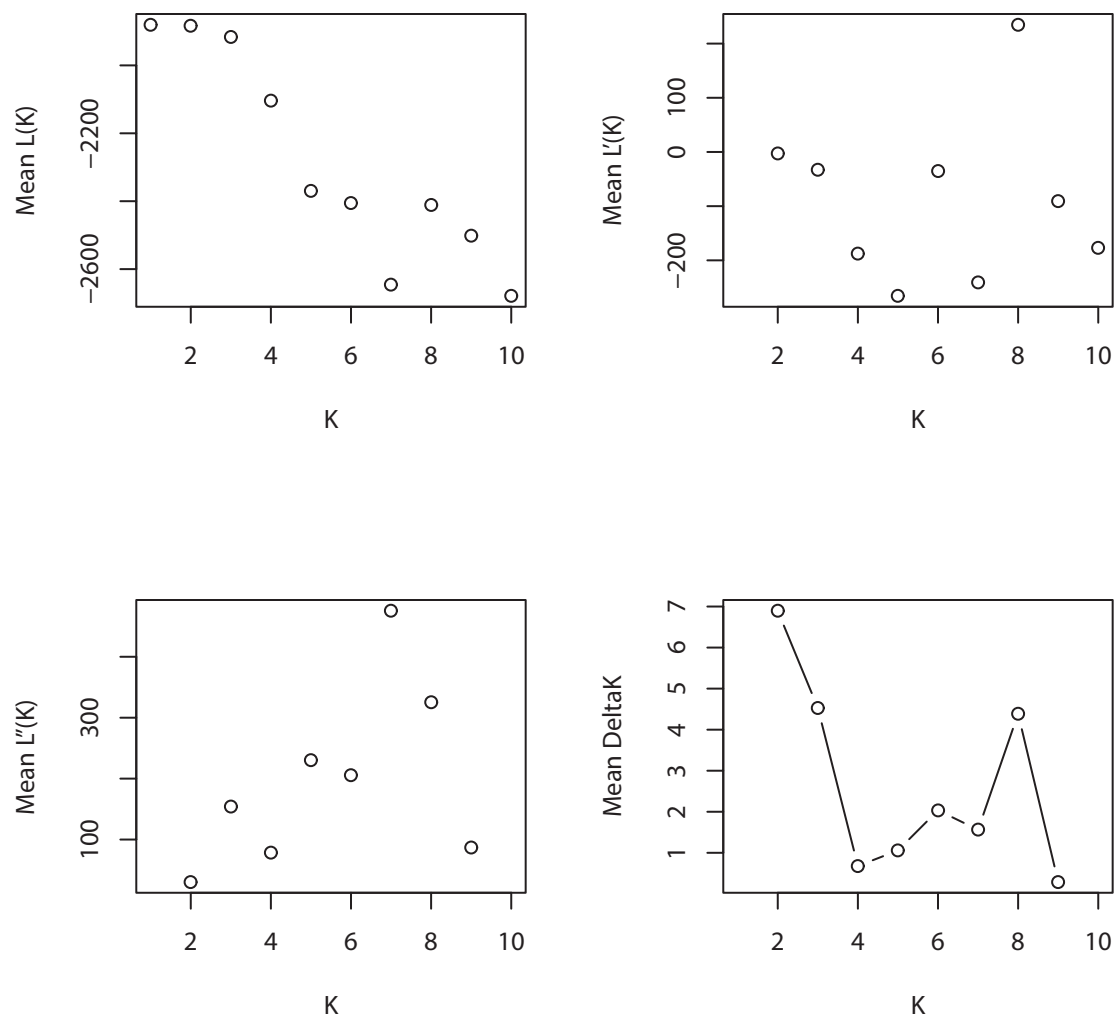

B

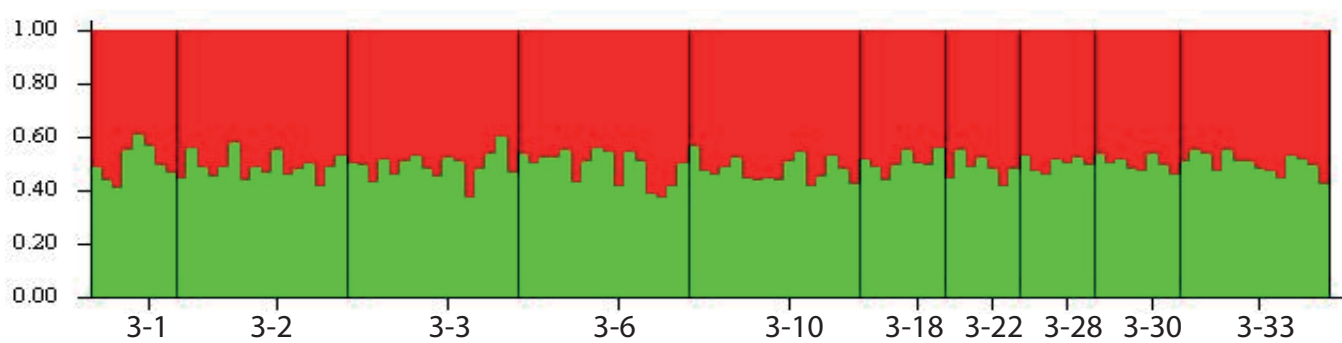

C

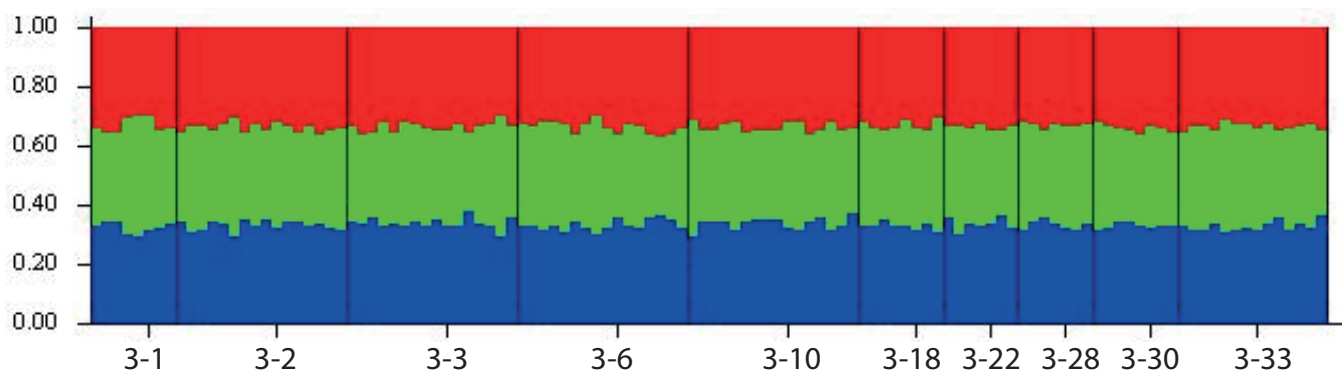

Supplement: Supplementary file 1 — Fig. S1 Results of structure analysis of microsatellite data. [file MEC-25-1595-s001.pdf]
